# Identification of beneficial symbiont candidates in commensalism as potential oral gatekeepers

**DOI:** 10.1128/spectrum.01588-25

**Published:** 2025-09-05

**Authors:** Soutaro Hanawa, Aoi Son, Tamotsu Kato, Yoshiyuki Matsuo, Takayuki Omae, Yuji Omori, Kyohei Yoshikawa, Koji Yamanegi, Kiichi Hirota, Hiroshi Ohno, Hideki Ogura, Satoshi Ishido, Kazuma Noguchi, Hiromitsu Kishimoto

**Affiliations:** 1 Department of Oral & Maxillofacial Surgery, School of Medicine, Hyogo Medical University 674409, Nishinomiya, Hyogo, Japan; 2 Department of Microbiology, School of Medicine, Hyogo Medical University 674409, Nishinomiya, Hyogo, Japan; 3 Laboratory for Intestinal Ecosystem, RIKEN Center for Integrative Medical Sciences 198286, Yokohama, Kanagawa, Japan; 4 Department of Human Stress Response Science, Institute of Biomedical Scienc, Kansai Medical University 314264, Hirakata, Osaka, Japan; 5 Department of Pathology, School of Medicine, Hyogo Medical University 674409, Nishinomiya, Hyogo, Japan; Nova Southeastern University, Fort Lauderdale, Florida, USA

**Keywords:** oral microbiota, beneficial symbiont, syntrophic interaction, genus *Rothia*, genus *Streptococcus*, probiotics

## Abstract

The basis of the development of oral cancer has been reported to be inflammation (e.g., periodontitis) caused by dysbiosis of the oral microbiota (i.e., a decrease in beneficial oral symbionts). Since a decrease in beneficial symbionts is connected to oral cancer, restoring these bacteria may help prevent it. Based on this, oral probiotics using beneficial oral symbionts are under development. Therefore, it is necessary to understand how beneficial oral symbionts are maintained in a healthy oral cavity. We evaluated a cohort consisting of 42 healthy volunteers and 39 oral cancer patients via 16S metagenomic analysis. Beneficial symbiont candidates were mined by comparing the oral microbiota of healthy volunteers with that of oral cancer patients, and the interaction mode among the beneficial symbiont candidates was further examined. Cancer patients exhibited decreased relative abundance of the genera *Rothia* and *Streptococcus*. In addition, both bacterial genera further decreased in advanced cancer, highlighting them as beneficial candidates. Furthermore, these two bacterial genera demonstrated a positive correlation in terms of relative abundance. *Rothia dentocariosa* isolated from a representative healthy volunteer was suggested to support the survival of *Streptococcus salivarius* through possible syntrophic interaction. Thus, we report a potential syntrophic interaction between *Streptococcus* spp. and *Rothia* spp. as a possible mechanism underlying oral health maintenance. Given that *S. salivarius* is currently employed as an oral probiotic, our findings provide insights into the development of probiotics for oral cancer.

**IMPORTANCE:**

Pathobiont candidates associated with oral cancer are currently being thoroughly investigated. However, it is not clear which bacteria and how their interactions contribute to preventing the development of oral cancer. In this report, we demonstrate for the first time the presence of a potential syntrophic interaction between *Rothia* spp. and *Streptococcus* spp., both of which were identified as beneficial symbiont candidates in the oral cavity.

## INTRODUCTION

Similar to other cancers, oral cancer is becoming increasingly common worldwide (1). The majority of oral cancer occurs on the tongue, and around 90% is squamous cell carcinoma (2). Excessive smoking and alcohol intake have been identified as an etiology of oral cancer (2). In addition, oral inflammation (e.g., chronic periodontitis) has been reported as an inducer of oral cancer (2, 3). Through technical advances in genomics, analysis of microbial community profiles in various niches has revealed significant microbial changes associated with diseases (i.e., loss of beneficial microbes and expansion of pathobionts), which is referred to as dysbiosis (4). In the oral cavity, periodontitis was demonstrated to be associated with a decreased frequency of some bacterial genera, including *Actinomyces*, *Rothia*, and *Streptococcus*, and an increased frequency of some bacterial genera, including *Fusobacteriu*m, and such a shift in the bacterial community was reported as an etiology in periodontitis (3, 5, 6). Since the onset of oral cancer is preceded by inflammation, dysbiosis of the bacterial community is proposed to cause oral cancer (7).

Given that dysbiosis is associated with a decrease in the frequency of beneficial symbionts, utilizing beneficial symbionts as probiotics could be a rational strategy to restore microbial homeostasis. To prevent oral inflammation, oral probiotics are currently being intensively investigated, with a strong emphasis on the genera *Lactobacillus* and *Bifidobacterium* (5). In addition, some of the *Streptococcus* spp. (e.g., K12) are already commercially available as oral probiotics (8). However, these probiotics have not consistently provided beneficial results, presumably due to differences in the host’s existing oral microbiomes of individuals (5). Therefore, additional investigations into probiotic candidates by searching dominant bacteria in the healthy oral cavity are underway (5, 8). Furthermore, since the healthy microbiome is maintained through complex microbial interactions, including cross-feeding (9), intake of single beneficial bacteria might not be sufficient. It may be necessary to identify beneficial symbionts with syntrophic interactions. Once beneficial symbionts with such an interaction are identified, these could stably/generally benefit the host as probiotics. This idea is similar to the one for the application of synbiotics, which are probiotics coupled with prebiotics (7). In the case of oral cancer, it is not clear what probiotics benefit the host by reshaping a disrupted oral microbiota (7, 10).

In light of the above, it is necessary to identify oral beneficial symbionts and understand how these microorganisms are maintained through bacterial interactions that prevent oral cancer. This study aimed not only to identify bacterial species that are reduced in oral cancer patients as compared to healthy individuals but also to investigate whether these species interact with each other to maintain a healthy oral environment.

## RESULTS

### Characterization of the subjects in analysis

To investigate beneficial symbiont candidates in the oral ecosystem, 39 oral cancer patients and 42 healthy volunteers were included in this analysis. Among the oral cancer patients, metastasis was detected in 10 cancer patients in the cervical lymph nodes by pathological examination during or after operation. The information regarding the participants included in this study is summarized in Table 1; Table S1. By comparing the microbiota of healthy volunteers with that of cancer patients with and without metastasis, potential beneficial symbionts in the oral ecosystem were explored.

**TABLE 1:** Clinical characteristics of subjects in this study^*a*^

Characteristics	Healthy volunteers	OSCC
Number of subjects	42	39
Status of metastasis	–^*b*^	29 (−) 10 (+)
Age (years)		
Range	48–86	38–87
Means ± SE	64.92 ± 1.469	69.31 ± 1.999
Gender		
Male	25 (60%)	23 (59%)
Female	17 (40%)	16 (41%)
Alcohol		
No	16 (38%)	16 (41%)
Yes	26 (61%)	23 (58%)
Smoking		
No	34 (80%)	25 (64%)
Yes	8 (19%)	14 (36%)

^*a*^ (−) without metastasis, (+) with metastasis.

^*b*^ “–”, not applicable.

### Characterization of the bacterial community in oral cancer patients

**Fig 1:**
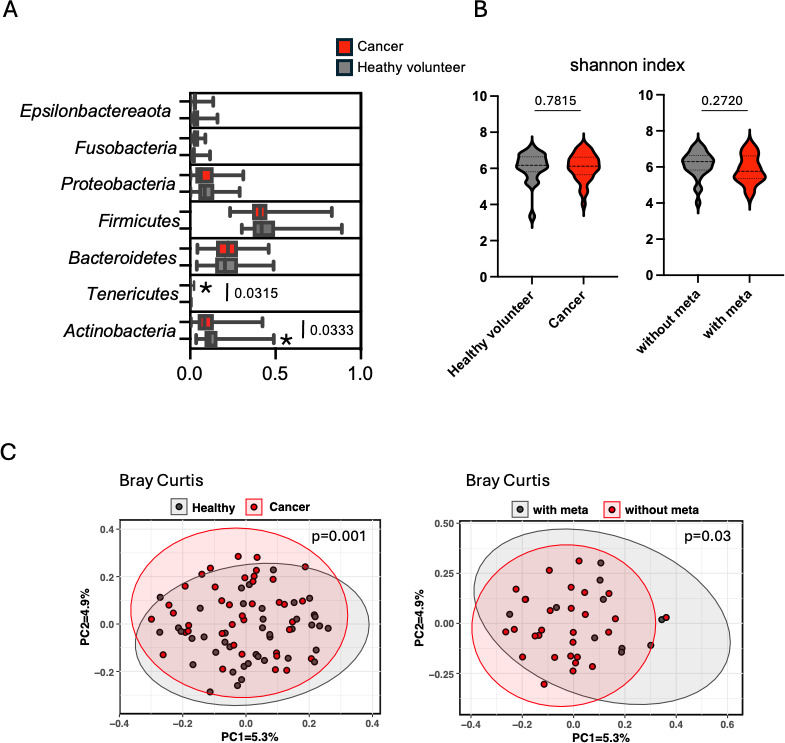
Characterization of oral microbiota in oral squamous cell carcinoma (OSCC). (**A**) The relative abundance of oral bacterial communities was shown at the phylum level in healthy volunteers and OSCC. (**B**) The alpha diversity indices were shown. The left panel showed the comparison between healthy volunteers and cancer patients. The right panel showed the comparison between the patients without metastasis and those with metastasis. Statistical analysis was performed by Mann-Whitney *U*-test. (**C**) The beta-diversity indices were shown. Same comparisons as in (B) were performed based on Bray-Curtis dissimilarity. Statistical analysis was performed by PERMANOVA.

The V1–V2 region amplicon of the 16S rRNA gene was analyzed by Miseq. The sequence reads of each sample that passed the criteria as described in the Materials and Methods section were subjected to analysis. The relative abundance of taxa was examined by the Qiime2 pipeline (11). In healthy volunteers, the most dominant bacterial phylum was Firmicutes (0.4378 ± 0.01671%) followed by Bacteroidetes (0.2148 ± 0.01573%), Actinobacteria (0.1303 ± 0.01156%), Proteobacteria (0.09726 ± 0.01080%), Epsilonbacteraeota (0.03484 ± 0.005340%), Fusobacteria (0.02693 ± 0.003678%), Spirochaetes (0.001271 ± 0.0005511%), and Tenericutes (0.001209 ± 0.0002910%) (Fig. 1A; Table S2). This trend was comparable to cancer patients (Fig. 1A). The alpha diversity indices (i.e., Shannon, Chao1, and Observed) did not demonstrate substantial differences between cancer patients and healthy volunteers (Fig. 1B; Fig. S1). Also, there were no significant differences between cancer patients with and without metastasis in the alpha diversity indices (Fig. 1B; Fig. S1B). In contrast, beta diversity indices revealed significant differences between cancer patients and healthy volunteers. Principal coordinate analysis demonstrated statistically different clustering in unweighted unifrac and Bray-Curtis distances between healthy volunteers and oral cancer patients (Fig. 1C; Fig. S2). Interestingly, there was a statistical difference in the beta diversity index between cancer patients with metastasis and cancer patients without metastasis (Fig. 1C). Unchanged alpha diversity indicated stable species richness, while beta diversity differences suggested community compositional shifts. These results indicated that community compositional shifts might be the biological basis of carcinogenesis and cancer progression in the oral cavity.

**Fig 2:**
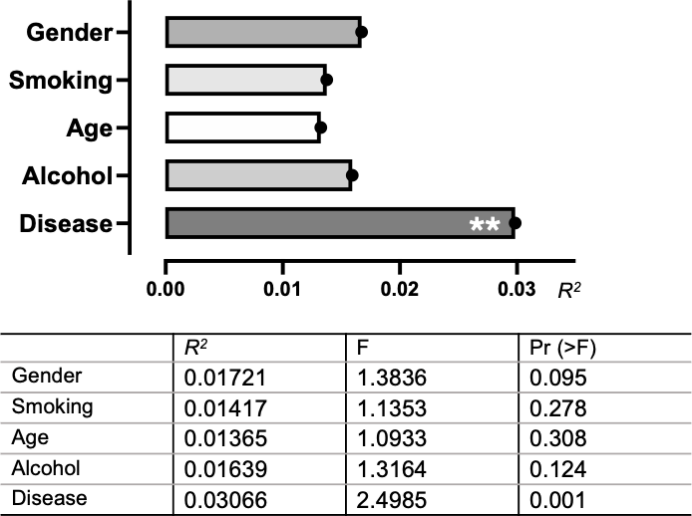
Analysis of the influence by subject factors on the oral bacterial community. Effect size (*R*^2^) of subject factors (e.g., gender, smoking, age, alcohol, and disease) on the oral bacterial community in the subjects was obtained using permutational multivariate analysis of variance (PERMANOVA) based on Bray-Curtis dissimilarity. The upper panel showed the summary of data shown in the lower panel. “**” indicates that Pr (>*F*) is 0.001.

The extent of influence by the demographics of the participant (i.e., gender, age, history of smoking, habit of drinking alcohol, and disease status [presence or absence of cancer]) on the bacterial community was examined using permutational multivariate analysis of variance (PERMANOVA). The value of *R*^2^, the effect size, obtained from disease status was highest among all examined demographic factors (Bray-Curtis *R*^2^ = 0.03066, *P* = 0.001) (Fig. 2). Furthermore, all examined demographic factors other than disease status did not show a significant effect on the oral bacterial community (Fig. 2). The influence of demographics of the participant on disease status was further assessed by chi-square and Fisher’s exact tests. All demographic factors were found not to significantly influence disease status, but the value of odds ratio from the history of smoking was highest (odds ratio = 2.38, 95% CI = 0.8621-6.811, *P* = 0.1331) (Table S10). Taken together, these data indicate the potential pathogenic contribution of community compositional shifts toward oral cancer, although the contribution of other demographic factors, such as smoking, could not completely be excluded in our cohort.

### Identification of genera *Rothia* and *Streptococcus* as beneficial symbiont candidates

**Fig 3:**
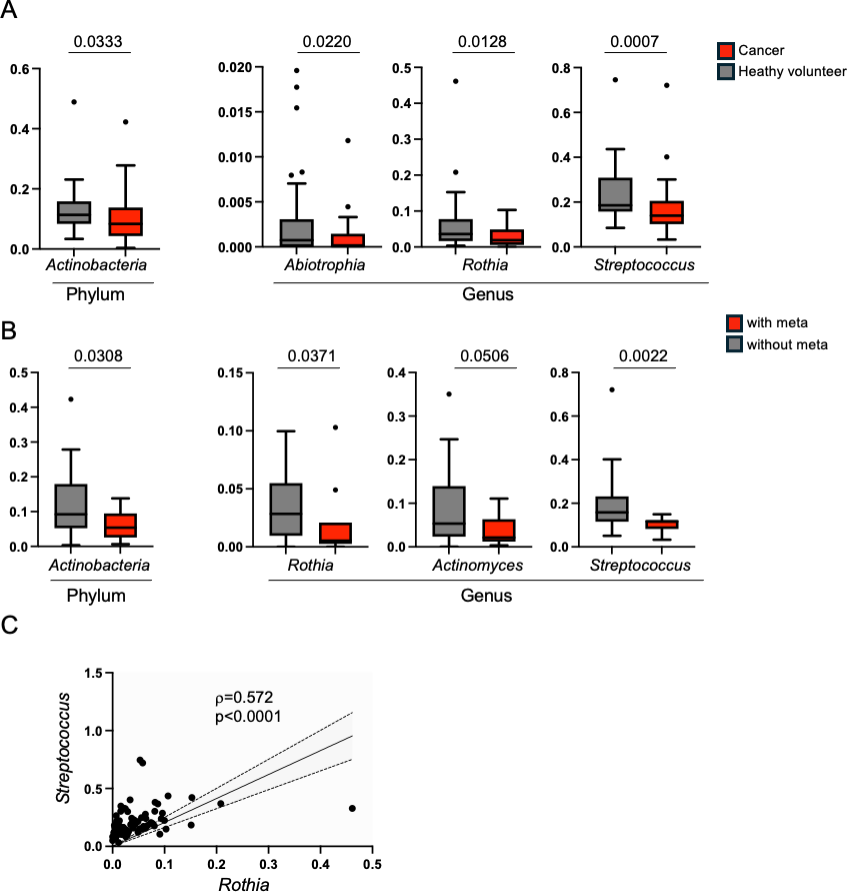
Identification of potential beneficial symbionts. (**A**) Bacterial communities whose relative abundance significantly decreased in the cancer patients were shown at the phylum level (left) and genus level (right). (**B**) Bacterial communities whose relative abundance progressively reduced along with metastasis were shown at the phylum level (left) and genus level (right). Each statistical analysis was performed by Mann-Whitney *U*-test (**A and B**). (**C**) Mining bacterial genera having a positive correlation with genus *Rothia* was performed by the Spearman’s Rank Correlation test. A representative bacterial genus that showed a positive correlation with genus *Rothia* was shown.

Next, we examined how the composition of the oral microbiota differs between cancer patients and healthy volunteers at the phylum and genus levels. At the phylum level, the frequency of *Actinobacteria* was shown to slightly but significantly decrease in cancer patients (Fig. 3A). At the genus level, cancer patients were found to have a decreased relative abundance of *Abiotrophia*, *Rothia*, and *Streptococcus*. Given that the genus *Rothia* belongs to the phylum *Actinobacteria*, we identified the genus *Rothia* as a beneficial symbiont candidate. Furthermore, we evaluated whether any bacterial populations decreased in parallel with the occurrence of metastasis in cancer patients. As shown in Fig. 3B, at the phylum level, *Actinobacteria* decreased in cancer patients with metastasis. Furthermore, at the genus level, *Rothia, Actinomyces,* and *Streptococcus* decreased (Fig. 3B; Table S4). Since both *Rothia* and *Streptococcus* were observed to decrease in cancer patients and further decrease in patients with metastasis, both genera were implied as beneficial symbiont candidates of the oral cavity.

### Identification of possible *Rothia-Streptococcus* interactions

Because bacterial syntrophic interactions have been previously demonstrated through exploring the co-occurrence of bacteria (12), we assessed whether any bacterial genera show co-occurrence with the genera *Rothia* or *Streptococcus* in the subjects. There were several bacterial genera showing a positive correlation with *Rothia* and *Streptococcus* (Fig. 3C; Table S5). Among them, *Rothia* showed a strong correlation with *Streptococcus* (ρ = 0.572, *P* < 0.0001) (Fig. 3C) and *Streptococcus_salivarius*_subsp_*thermophilus* (*ρ* = 0.4368, *P* < 0.0001) (Table S5). These results implied that the genera *Rothia* and *Streptococcus* closely interact with each other through a syntrophic interaction.

### Negative correlation of potential beneficial symbionts with pathobiont candidates

**Fig 4:**
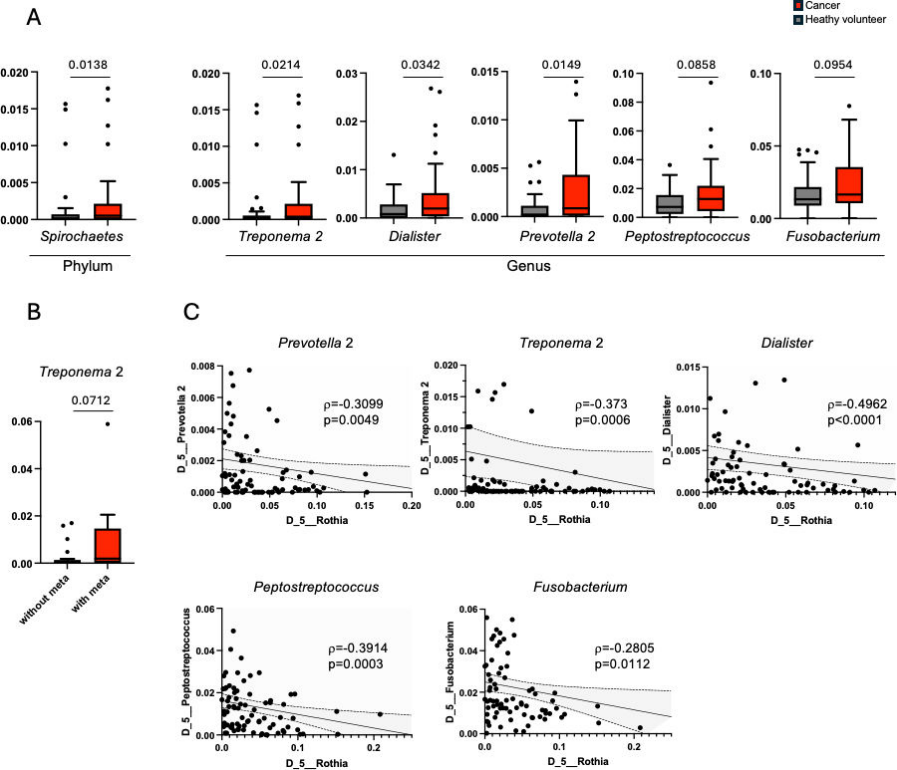
Identification of potential pathobionts. (**A**) Bacterial communities whose relative abundance increased in the cancer patients were shown at the phylum level (left) and genus level (right). (**B**) Bacterial communities whose relative abundance showed increasing tendency along with metastasis at the genus level were shown. Each statistical analysis was performed by Mann-Whitney *U*-test (**A and B**). (**C**) Negative correlation of genus *Rothia* with five pathobiont candidates in terms of frequency was shown. Analysis was performed by the Spearman’s rank correlation test.

Given that the relative abundance of beneficial symbionts might negatively correlate with pathobionts in the bacterial community (4), we next assessed the relationship between the genus *Rothia* and pathobiont candidates in our cohort. Pathobiont genera candidates, including *Treponema 2*, *Alloprevotella*, *Dialister*, *Gemella*, *Parvimonas*, and *Prevotella 2*, were identified because of their increased frequency in cancer patients (Fig. 4A; Table S3). Among them, *Treponema* 2 could be a strong candidate, because the phylum *Spirochaetes,* which the genus *Treponema* 2 belongs to, also increased in cancer patients (Fig. 4A; Tables S2 and S3) and we also observed an increasing trend of the genus *Treponema* 2 in oral cancer patients with metastasis (Fig. 4B). In addition, the genera *Peptostreptococcus* and *Fusobacterium* are also pathobiont candidates, as these genera also had an increasing trend observed in oral cancer patients (Fig. 4A; Table S3). Examination of the relationship between the genus *Rothia* and these pathobiont candidates demonstrated that the genus *Rothia* negatively correlated with *Prevotella 2*, *Treponema 2*, *Dialister*, *Peptostreptococcus*, and *Fusobacterium* (Fig. 4C; Table S5). These bacterial genera also negatively correlated with *Streptococcus* (Table S5). Thus, the relative abundance of *Rothia* and *Streptococcus* negatively correlated with several pathobiont candidates, further highlighting both the *Rothia* and *Streptococcus* genera as beneficial symbiont candidates in our cohort.

### Identification of bacterial interactions with possible syntrophy between the genera *Rothia* and *Streptococcus*

**Fig 5:**
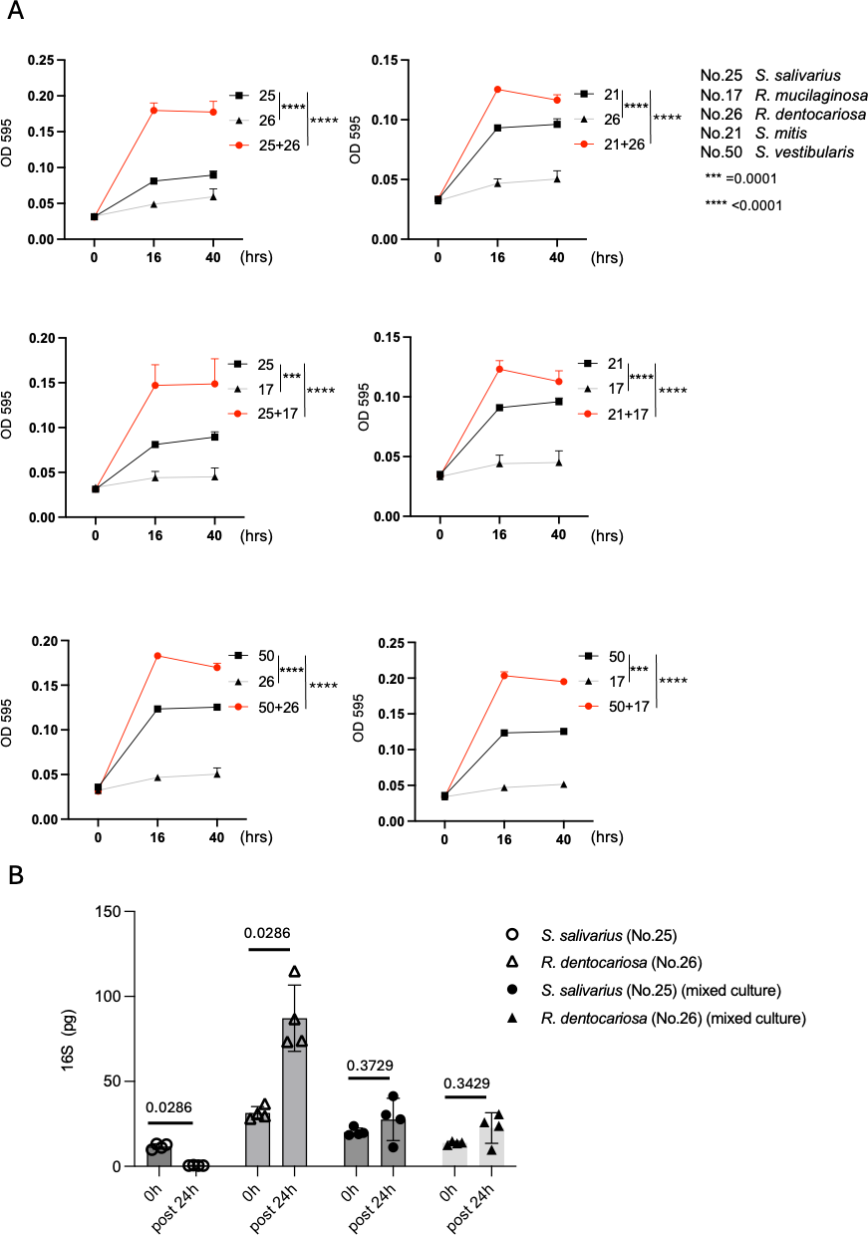
Bacterial interaction between the genera *Rothia* and *Streptococcus*. (**A**) The cellular status of unmixed bacteria and mixed bacteria was shown as OD 595 (nm) values during cultivation. The information on bacteria in each set of experiment was shown in the right upper side. Each statistical analysis was performed by one-way ANOVA and adjusted *P* values were shown. (**B**) Among the bacteria analyzed in (A), *R. dentocariosa* and *S. salivarius* were further examined. The amount of 16S of each bacterium in each set of cultivation was measured by 16 rRNA qPCR as shown in the Materials and Methods section. The status of each bacterium was examined by comparing the value at pre-cultivation (0 h) with the value after 24 h cultivation in defined medium. Statistical analysis was performed by Mann-Whitney *U*-test, and each value was shown.

Potential syntrophic interactions between the genera *Rothia* and *Streptococcus* have been identified; thus, we further examined this possibility by cultivating the bacteria isolated from a representative healthy subject. *Rothia mucilaginosa* and *Rothia dentocariosa* were identified as representatives of *Rothia* spp. and *Streptococcus salivarius*, *Streptococcus mitis,* and *Streptococcus vestibularis* were identified as representatives of *Streptococcus* spp. in the subjects whose frequency of *Rothia* was relatively high (Table S6), and these representative bacteria were isolated from a representative healthy subject. In a defined culture medium (M9 containing 2% glucose as a carbohydrate), all isolated *Rothia* spp. exhibited low OD_595_ values as compared with the isolated *Streptococcus* spp. (Fig. 5A). To examine the possibility of a syntrophic interaction between *Rothia* spp. and *Streptococcus* spp., we compared the OD_595_ values under single culture with those under mixed culture. Interestingly, the OD_595_ values under mixed culture were higher than those under single culture. The same result was observed in all combinations (Fig. 5A).

**Fig 6:**
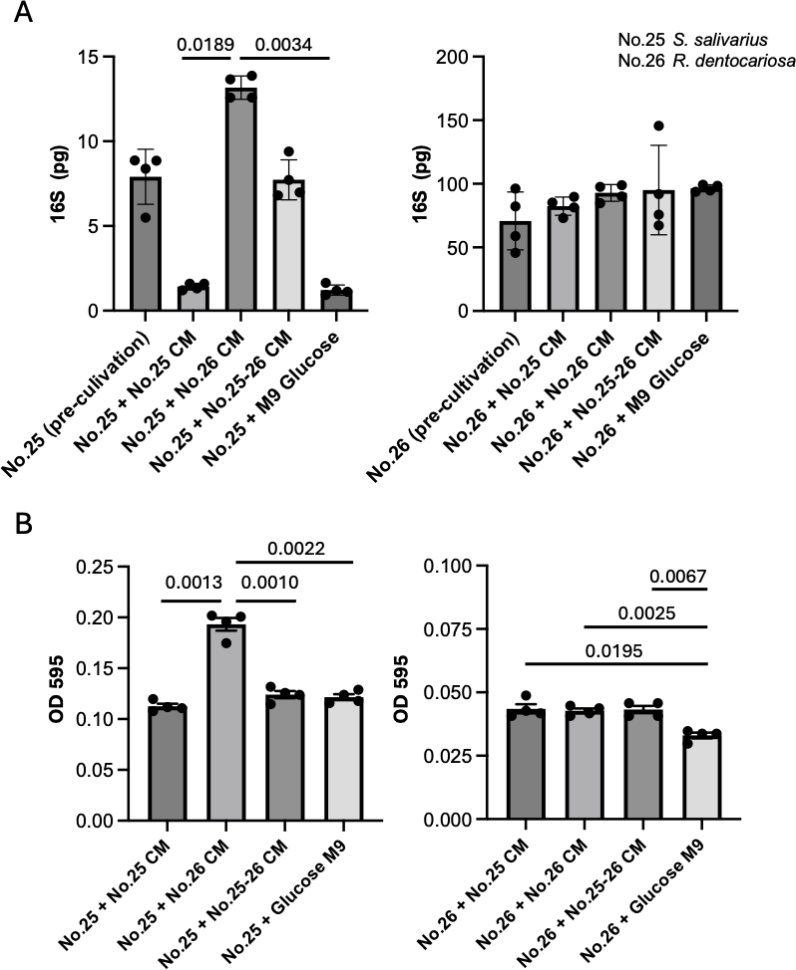
Possible syntrophic interaction between *R. dentocariosa* and *S. salivarius*. (**A**) (Left) *S. salivarius* (No. 25) was cultivated with the indicated conditioned medium (CM) and monitored for 24 h. Each 16S value was compared among the indicated four experimental sets and the pre-cultivation set. (Right) The results of *R. dentocariosa* (No.26) were shown in the left panel. Statistical analysis was performed by one-way ANOVA. (**B**) Same experiments as in (**A**) were performed by measuring OD (595 nm). Each OD value was compared among the indicated four experimental sets. Statistical analysis was performed by one-way ANOVA. Each value was shown when judged as significant.

Since the OD_595_ value represents several cellular activities (e.g., cell growth and polysaccharide production), the status of cell growth was estimated by qPCR for the 16S rRNA gene. Surprisingly, the results of the 16S rRNA qPCR demonstrated that *S. salivarius* could not survive efficiently in this defined condition, but *R. dentocariosa* was able to survive (Fig. 5B). Calculations for *S. salivarius* in the mixed culture indicated that *R. dentocariosa* provided biological support in terms of survival, because the reduction of *S. salivarius* was rescued by co-cultivation (Fig. 5B). This result suggested the presence of cross-fed metabolites derived from *R. dentocariosa* for *S. salivarius*. To test this theory, we examined the ability of the conditioned medium from *R. dentocariosa* to support the survival of *S. salivarius*. As shown in Fig. 6A, conditioned medium of *R. dentocariosa* supported the survival of *S. salivarius*. In contrast, there was no similar biological effect from the conditioned medium derived from *S. salivarius* on *R. dentocariosa* (Fig. 6A). These results were verified by measuring the OD_595_ values. Consistent with the results from the 16S rRNA qPCR, conditioned medium of *R. dentocariosa* increased the OD_595_ values of *S. salivarius*. However, conditioned medium of *S. salivarius* did not influence the OD_595_ values of *R. dentocariosa* (Fig. 6B). Taken together, these results indicate the presence of a possible syntrophic interaction between *R. dentocariosa* and *S. salivarius,* and this bacterial relationship was thought to be commensalism (9).

## DISCUSSION

In this report, we demonstrated two beneficial symbiont candidates with a possible syntrophic interaction. *Rothia* spp. was suggested to support the survival of *Streptococcus* spp. by a unidirectional interaction for oral health. It is still not clear how helpful oral bacteria are maintained in a healthy oral cavity; therefore, this report gives new evidence for future research in the field. Given that normalization of the oral microbiota is a promising approach against oral cancer, this information could support further experiments investigating how *Rothia-Streptococcus* interactions contribute to host fitness.

This report could be informative for the development of the next generation of probiotics, especially since *Streptococcus* spp. has already been utilized as an oral probiotic. Several reports have demonstrated that *Streptococcus* K12, a strain of *S. salivarius,* has killing ability against oral pathobionts (e.g., *Fusobacteria*) through producing bacteriocin-like inhibitory substances (8). Intake of *Rothia* spp. with K12 might stabilize the benefit from K12 through possible syntrophic interactions. In addition, *Rothia* itself was reported to benefit the host by suppressing pathobionts through nitrite production (13). Although confirmation of these data might be required, it would still be valuable to examine the effects of coadministration of *Rothia* spp. with *Streptococcus* spp.

Although there is a limitation in identifying detailed bacterial taxonomic levels in some sequencing systems (e.g., MiSeq), the data regarding bacterial communities have been robustly accumulated. However, the nature of dysbiosis in oral cancer has been varied among the reports, which might be due to the diverse lifestyles and races (14) or different specifications of the sequencing analyzer. Nonetheless, data similar to that presented in the present study have been reported; one study demonstrated that both *Rothia* and *Streptococcus* significantly decreased in oral cancer patients (15), and another Japanese study demonstrated that *Rothia* spp. decreased in oral cancer patients, with the extent of decrease correlating with disease progression (16). In addition, either *Rothia* spp. or *Streptococcus* spp. was reported to decrease in oral cancer patients across the countries (17–19). Further investigation is required, but *Rothia-Streptococcus* interactions might frequently play a crucial role in the maintenance of oral health.

Bacteria physiologically interact with each other, presumably toward host fitness. Previous studies have shown that *Bacteroides* spp. support butyrate production from butyrate-producing bacteria by generating cross-fed metabolites (i.e., polysaccharide breakdown products) through a polysaccharide utilization locus (20, 21). In this study, we found supportive evidence indicating that *S. salivarius* depends on *R. dentocariosa* to survive (Fig. 5A). This support might be achieved by exhaustion of *R. dentocariosa*, because co-cultivation decreased the growth of *R. dentocariosa* (Fig. 5B). A prior study showed that *R. mucilaginosa* produces enterobactin, a metal-chelating siderophore that can support the growth of *S. salivarius* by examination of laboratory bacteria strains; accordingly, providing additional ions to the other bacteria by enterobactin was reported as the mechanism of the support (22). If this is accurate, then the contribution of Mg^2+^ to our results might be suggested, because our co-culture experiment was performed in the presence of MgSO_4_. Also, it was reported that the conditioned medium of cultured *R. mucilaginosa* showed anti-inflammatory effects through the inhibition of the NF-kB pathway *in vitro* (23). Thus, although further experiments are required, *Rothia* spp. might provide several cross-fed metabolites, and these products might be applicable as prebiotics.

We reported additional informative data regarding oral beneficial symbiont candidates. For example, the genus *Abitrophia* was shown to decrease in oral cancer patients (Fig. 3A). However, since its relative abundance was quite low in healthy subjects (0.004447 ± 0.001484%) as compared with the genera *Rothia* and *Streptococcus* (Table S3), its contribution might be limited. In contrast, the genus *Actinomyces,* of which the frequency is equivalent to the genus *Rothia,* was found to decrease in oral cancer patients with metastasis with marginal significance (*P* = 0.0506) (Fig. 3B). Interestingly, *Actinomyces* did not decrease in oral cancer patients as compared with healthy volunteers (Table S3), suggesting that *Actinomyces* plays a role in the progression of oral cancer, but not to the initiation of oral cancer. Given that the genus *Actinomyces* was reported to decrease in oral cancer patients (15), it may contain beneficial symbionts. In addition to the genus *Actinomyces*, *Granulicatella* might be another beneficial genus because of a strong correlation with the genera *Rothia* and *Streptococcus* (Fig. 3C) (Table S5) and its decreasing trend in cancer patients (Table S3). Thus, it might be of significance to investigate these bacteria as future probiotics.

There are several limitations in this study. First, since the analysis of the bacterial community is based on 16S rRNA sequencing, but not shotgun metagenomics, there is the possibility of false genus or species-level assignments. Second, we reported that the *Rothia-Streptococcus* interaction potentially contributes to oral health, but since this argument was based on the comparative analysis of bacterial community between healthy volunteers and oral cancer patients, functional and clinical validation are both required. Third, we demonstrated a possible syntrophic interaction between *Rothia* spp. and *Streptococcus* spp. by measuring the OD_595_ and qPCR experiments, but since these are proxy measurements, further metabolic validation is required to show syntrophic cross-feeding.

We reported a possible helpful interaction between genus *Rothia* and genus *Streptococcus*. We believe that this report is valuable as early evidence for the development of these genera as oral probiotics, particularly because all of the data were derived from human clinical samples.

## MATERIALS AND METHODS

### Patient enrollment and sample collection

This study was reviewed and approved by the Ethics Committee of Hyogo Medical University at Nishinomiya (No. 3882). Patients diagnosed with oral cancer in Hyogo Medical University Hospital from September 2021 through July 2023 were added to a list along with the patient’s information. Exclusion criteria were as follows: (i) patients with a history of antibiotic and immune modulatory treatment 2 weeks prior to diagnosis; and (ii) patients who have previously undergone intensive treatment (e.g., surgery with radiation therapy) for oral cancer. The patients judged as eligible by both medical records and specific questionnaires were enrolled based on receiving written informed consent for their contribution to this study. Saliva was collected from the subjects 1–2 weeks after enrollment using Salivette cotton swabs (SARSTEDT AG & Co. Nümbrecht, Germany). The cotton swabs within the Salivette Kit were centrifuged to harvest all microbes collected with the saliva. The total DNA was extracted from the pellets via phenol-chloroform. The quality and quantity of the extracted DNA were assessed by agarose gel electrophoresis and measurement of the ratio of 260/280 by NanoDrop (Thermo Fisher Scientific Inc., CA, USA). DNA that passed quality confirmation was utilized in future steps.

### 16S rRNA amplicon sequencing, data bioinformatics, and statistical analysis

The V1–V2 variable region (27Fmod and 338R) of the 16S rRNA gene was amplified from 50 ng of extracted DNA by PCR. The primers used are as follows:

27Fmod: 5′-TCGTCGGCAGCGTCAGATGTGTATAAGAGACAGAGRGTTTGATYMTGGCTCAG-3′

338R: 5′-GTCTCGTGGGCTCGGAGATGTGTATAAGAGACAGTGCTGCCTCCCGTAGGAGT-3′ (24). The PCR amplicons were barcoded by a Nextera XT Index Kit v2 and analyzed by Miseq (Illumina, Inc., Dan Diego, CA, USA). The obtained sequences were filtered by Trimmomatic software (http://www.usadellab.org/cms/?page=trimmomatic), followed by analysis with the Qiime2 pipeline: qiime2-amplicon-2024.2 (11). The sequencing reads underwent quality filtering, denoising, and chimera removal, and the processed/selected ones were merged using the DADA2 package to construct a feature table of ASV (25). Classification of the feature sequences was conducted by a feature-classifier plug-in based on silva-138-99-nb-classifier.qza. All 16S rRNA gene data sets were deposited in the DDBJ Sequence Read Archive (accession number: PRJDB35665). The relative abundance of each taxon in the subjects was calculated by a taxon plug-in at the phylum through genus level (Tables S8 and S9).

Differences in the relative abundance of each taxon between groups (e.g., healthy vs oral cancer) were statistically examined by nonparametric *t*-tests (Mann-Whitney *U*-test). Furthermore, to assess differences in the bacterial community between healthy and oral cancer subjects, or between oral cancer without metastasis and oral cancer with metastasis, alpha and beta diversity analyses were performed using a diversity plug-in. A molecular phylogenetic tree of the samples was constructed using a phylogeny plug-in, and the number of sequence reads subjected to analysis (e.g., sampling depth number) was determined by referring to an alpha-rarefaction curve from all samples. Based on the determined sampling depth, alpha and beta diversity indices were calculated. Statistical analysis of alpha and beta diversity indices was performed by Mann-Whitney *U*-test and PERMANOVA, respectively. The data utilized for statistical analysis of the alpha diversity are shown in Table S11.

The influence of the demographics of each subject, including disease status, gender, age, smoking history, and alcohol drinking habit, on their oral bacterial community was statistically analyzed by PERMANOVA, as performed previously (15). Disease status was discriminated based on the presence of cancer. Regarding age, subjects were divided at 60 years of age. A history of smoking or alcohol consumption was marked as “positive.” The data set of the relative abundance of each taxon, which was obtained through the qiime2 pipeline (Table S7), was subjected to analysis using the adonis2 in Vegan package. A Bray-Curtis index was employed in this analysis. In addition, the influences of each subject’s demographics on disease status were examined by a chi-square and Fisher’s exact tests, which was performed using Prism 9.2.

### Exploration of beneficial symbiont candidates and their interactions

First, bacterial genera with a relative abundance statistically decreased in cancer patients were selected. Among these, bacteria with a relative abundance that was further decreased with metastatic progression were selected as beneficial symbiont candidates. Next, bacterial genera with a relative abundance that positively correlated with beneficial symbiont candidates were explored by calculating pairwise Spearman’s rank correlations. A *ρ* value greater than 0.3 and a *P* value less than 0.05 were considered to indicate significant positive correlation as reported previously (19). All calculations were performed using Prism 9.2.

### Examination of bacterial interactions in *vitro*

All *in vitro* experiments with isolated bacteria were approved by the Biosafety Committee of Hyogo Medical University (25-067P). Before starting the isolation of any bacteria, dominant bacteria belonging to the genera *Rothia* and *Streptococcus* in the oral cavity of subjects whose frequency of *Rothia* was relatively high (Table S6) were examined by MinION nanopore sequencing (Oxford Nanopore Technologies, Oxford, UK). DNA samples were shared for the analysis of Miseq. Then 1.5 kb-long reads derived from 16S rRNA were obtained by PCR with KOD-plus DNA polymerase (TOYOBO, Osaka, Japan), and barcoded amplicons were analyzed (26). Dominant species within the bacterial genera were determined based on approximately 10,000 of the 1.5 kb-long reads from representative subjects. As a result, *Rothia mucilaginosa* was detected as a dominant species, followed by *R. dentocariosa* and *R. aeria* (Table S6). In two healthy volunteers, *S. salivarius* was detected as a major species in the genus *Streptococcus* (Table S6). Saliva samples from a representative healthy subject were cultured on a KBM Columbia CA sheep blood agar plate (KOHJIN BIO, Saitama, Japan) for 2 days under aerobic conditions, and the colonies of the dominant species were determined by Bruker MALDI Biotyper (Hitachi, Tokyo, Japan). All isolated bacteria were stocked in skim milk.

Isolated *Rothia* spp. and *Streptococcus* spp. were cultivated in modified GAM broth (SHIMADZU, Kyoto, JA) until they entered the exponential phase. The cultures were centrifuged, washed with defined M9 medium (3 g Na_2_HPO_4_, 1.5 g KH_2_PO_4_, 0.5 g NH_4_Cl, 0.25 g NaCl, and 1.5 mg CaCl_2_/L) containing 2% glucose, 1 mM MgSO_4_⋅7H_2_O, 0.00005% vitamin B1, 0.1% casamino acid, and adjusted to an optical density at 595 nm (OD_595_) of 0.03 with same culture medium. *Rothia* spp. and *Streptococcus* spp. were mixed at a 1:1 ratio as volume. Mixed bacteria, unmixed *Rothia* spp., and unmixed *Streptococcus* spp. were subjected to examination of cellular status by monitoring the OD_595_ in a 96-well culture plate. The OD_595_ of all three bacteria samples was initially fixed to 0.03 and then measured for 48 h using a BIO-RAD microplate reader (BIO-RAD, Hercules, CA, USA). The differences in the cellular status among the three groups were statistically examined based on the OD value by a one-way ANOVA.

To estimate interbacterial interactions in terms of bacterial growth, the growth status of each indicated bacterium was estimated by 16S rRNA qPCR with KAPA SYBR Fast qPCR kit (KAPA BYOSYSTEMS, Inc., Wilmington, MA, USA) under single (unmixed) or mixed cultivation (27). DNA was extracted from each culture by phenol-chloroform extraction and subjected to 16S rRNA qPCR analysis. The level of the 16S rRNA gene in each mixed culture was calculated as previously reported (20). The total amount obtained by 16S rRNA qPCR was multiplied by the relative abundance of each bacterium in the mixed culture, which was determined by MinION nanopore sequencing (26). The data were statistically analyzed by a Mann-Whitney *U*-test.

The ability of the conditioned medium to activate *R. dentocariosa* or *S. salivarius* was tested as follows. The conditioned medium from each of the three sets (i.e., mixed bacteria, unmixed *R. dentocariosa*, and unmixed *S. salivarius*) was collected after culturing for 24 h in defined medium, filtered, and added to a culture of *S. salivarius* or *R. dentocariosa*. Fresh defined medium was included as a control for the conditioned medium. Before adding the conditioned medium, *R. dentocariosa* and *S. salivarius* were cultivated until they reached the exponential phase of growth. The cultures were then washed with defined medium and resuspended to an OD_595_ of 0.03 with the same media. The activation status of *R. dentocariosa* or *S. salivarius* was examined by 16S rRNA qPCR, in addition to monitoring the OD_595_ values for 24 h. The results were statistically analyzed by a one-way ANOVA.

## Data Availability

All 16S rRNA gene data sets were deposited in the DDBJ Sequence Read Archive (accession number: PRJDB35665).

## SUPPLEMENTAL MATERIAL

Fig. S1 spectrum.01588-25-s0001.tiff Characterization of oral microbiota in OSCC.

Fig. S2 spectrum.01588-25-s0002.tiff Characterization of oral microbiota in OSCC.

Supplemental tables spectrum.01588-25-s0003.xlsx Tables S1 to S11.

ASM does not own the copyrights to Supplemental Material that may be linked to, or accessed through, an article. The authors have granted ASM a non-exclusive, world-wide license to publish the Supplemental Material files. Please contact the corresponding author directly for reuse.

## References

[1] Lekshmi Priya KS, Maheswary D, Ravi SSS, Leela KV, Lathakumari RH, Malavika G. 2025. The impact of probiotics on oral cancer: mechanistic insights and therapeutic strategies. Oral Oncol Rep 13:100715. doi:10.1016/j.oor.2025.100715

[2] Wan Mohd Kamaluddin WNF, Rismayuddin NAR, Ismail AF, Mohamad Aidid E, Othman N, Mohamad NAH, Arzmi MH. 2020. Probiotic inhibits oral carcinogenesis: a systematic review and meta-analysis. Arch Oral Biol 118:104855. doi:10.1016/j.archoralbio.2020.104855 32801092

[3] Kleinstein SE, Nelson KE, Freire M. 2020. Inflammatory networks linking oral microbiome with systemic health and disease. J Dent Res 99:1131–1139. doi:10.1177/0022034520926126 32459164 PMC7443998

[4] Petersen C, Round JL. 2014. Defining dysbiosis and its influence on host immunity and disease. Cell Microbiol 16:1024–1033. doi:10.1111/cmi.12308 24798552 PMC4143175

[5] Del Pilar Angarita-Díaz M, Fong C, Medina D. 2024. Bacteria of healthy periodontal tissues as candidates of probiotics: a systematic review. Eur J Med Res 29:328. doi:10.1186/s40001-024-01908-2 38877601 PMC11177362

[6] Curtis MA, Zenobia C, Darveau RP. 2011. The relationship of the oral microbiotia to periodontal health and disease. Cell Host Microbe 10:302–306. doi:10.1016/j.chom.2011.09.008 22018230 PMC3216488

[7] Mohd Fuad AS, Amran NA, Nasruddin NS, Burhanudin NA, Dashper S, Arzmi MH. 2023. The mechanisms of probiotics, prebiotics, synbiotics, and postbiotics in oral cancer management. Probiotics Antimicrob Proteins 15:1298–1311. doi:10.1007/s12602-022-09985-7 36048406 PMC9434094

[8] Tagg JR, Harold LK, Jain R, Hale JDF. 2023. Beneficial modulation of human health in the oral cavity and beyond using bacteriocin-like inhibitory substance-producing streptococcal probiotics. Front Microbiol 14:1161155. doi:10.3389/fmicb.2023.1161155 37056747 PMC10086258

[9] Culp EJ, Goodman AL. 2023. Cross-feeding in the gut microbiome: ecology and mechanisms. Cell Host Microbe 31:485–499. doi:10.1016/j.chom.2023.03.016 37054671 PMC10125260

[10] Kumar PDM, Poorni S, Ranganathan K, Aswath Narayanan MB. 2023. Probiotics: are they a game changer in oral cancer research and management? Cancer Res Stat Treat 6:623–624. doi:10.4103/crst.crst_329_23

[11] Bolyen E, Rideout JR, Dillon MR, Bokulich NA, Abnet CC, Al-Ghalith GA, Alexander H, Alm EJ, Arumugam M, Asnicar F, et al.. 2019. Reproducible, interactive, scalable and extensible microbiome data science using QIIME 2. Nat Biotechnol 37:852–857. doi:10.1038/s41587-019-0209-9 31341288 PMC7015180

[12] Ruaud A, Esquivel-Elizondo S, Cuesta-Zuluaga J, Waters JL, Angenent LT, Youngblut ND, Ley RE. 2020. Syntrophy via interspecies H2 transfer between Christensenella and Methanobrevibacter underlies their global cooccurrence in the human gut. mBio 11:e03235-19. doi:10.1128/mbio.03235-19 32019803 PMC7002349

[13] West SR, Suddaby AB, Lewin GR, Ibberson CB. 2024. Rothia. Trends Microbiol 32:720–721. doi:10.1016/j.tim.2024.03.009 38580605

[14] Yu X, Shi Y, Yuan R, Chen Z, Dong Q, Han L, Wang L, Zhou J. 2023. Microbial dysbiosis in oral squamous cell carcinoma: a systematic review and meta-analysis. Heliyon 9:e13198. doi:10.1016/j.heliyon.2023.e13198 36793959 PMC9922960

[15] Cai L, Zhu H, Mou Q, Wong PY, Lan L, Ng CWK, Lei P, Cheung MK, Wang D, Wong EWY, Lau EHL, Yeung ZWC, Lai R, Meehan K, Fung S, Chan KCA, Lui VWY, Cheng ASL, Yu J, Chan PKS, Chan JYK, Chen Z. 2024. Integrative analysis reveals associations between oral microbiota dysbiosis and host genetic and epigenetic aberrations in oral cavity squamous cell carcinoma. NPJ Biofilms Microbiomes 10:39. doi:10.1038/s41522-024-00511-x 38589501 PMC11001959

[16] Takahashi Y, Park J, Hosomi K, Yamada T, Kobayashi A, Yamaguchi Y, Iketani S, Kunisawa J, Mizuguchi K, Maeda N, Ohshima T. 2019. Analysis of oral microbiota in Japanese oral cancer patients using 16S rRNA sequencing. J Oral Biosci 61:120–128. doi:10.1016/j.job.2019.03.003 31109865

[17] Perera M, Al-Hebshi NN, Perera I, Ipe D, Ulett GC, Speicher DJ, Chen T, Johnson NW. 2018. Inflammatory bacteriome and oral squamous cell carcinoma. J Dent Res 97:725–732. doi:10.1177/0022034518767118 29630846

[18] Schmidt BL, Kuczynski J, Bhattacharya A, Huey B, Corby PM, Queiroz ELS, Nightingale K, Kerr AR, DeLacure MD, Veeramachaneni R, Olshen AB, Albertson DG. 2014. Changes in abundance of oral microbiota associated with oral cancer. PLoS One 9:e98741. doi:10.1371/journal.pone.0098741 24887397 PMC4041887

[19] Ganly I, Yang L, Giese RA, Hao Y, Nossa CW, Morris LGT, Rosenthal M, Migliacci J, Kelly D, Tseng W, Hu J, Li H, Brown S, Pei Z. 2019. Periodontal pathogens are a risk factor of oral cavity squamous cell carcinoma, independent of tobacco and alcohol and human papillomavirus. Int J Cancer 145:775–784. doi:10.1002/ijc.32152 30671943 PMC6554043

[20] Feng J, Qian Y, Zhou Z, Ertmer S, Vivas EI, Lan F, Hamilton JJ, Rey FE, Anantharaman K, Venturelli OS. 2022. Polysaccharide utilization loci in Bacteroides determine population fitness and community-level interactions. Cell Host Microbe 30:200–215. doi:10.1016/j.chom.2021.12.006 34995484 PMC9060796

[21] Rodriguez-Castaño GP, Dorris MR, Liu X, Bolling BW, Acosta-Gonzalez A, Rey FE. 2019. Bacteroides thetaiotaomicron starch utilization promotes quercetin degradation and butyrate production by Eubacterium ramulus Front Microbiol 10:1145. doi:10.3389/fmicb.2019.01145 31191482 PMC6548854

[22] Uranga CC, Arroyo P, Duggan BM, Gerwick WH, Edlund A. 2020. Commensal oral Rothia mucilaginosa produces enterobactin, a metal-chelating siderophore. mSystems 5:e00161-20. doi:10.1128/mSystems.00161-20 32345739 PMC7190385

[23] Rigauts C, Aizawa J, Taylor SL, Rogers GB, Govaerts M, Cos P, Ostyn L, Sims S, Vandeplassche E, Sze M, Dondelinger Y, Vereecke L, Van Acker H, Simpson JL, Burr L, Willems A, Tunney MM, Cigana C, Bragonzi A, Coenye T, Crabbé A. 2022. Rothia mucilaginosa is an anti-inflammatory bacterium in the respiratory tract of patients with chronic lung disease. Eur Respir J 59:2101293. doi:10.1183/13993003.01293-2021 34588194 PMC9068977

[24] Kim SW, Suda W, Kim S, Oshima K, Fukuda S, Ohno H, Morita H, Hattori M. 2013. Robustness of gut microbiota of healthy adults in response to probiotic intervention revealed by high-throughput pyrosequencing. DNA Res 20:241–253. doi:10.1093/dnares/dst006 23571675 PMC3686430

[25] Callahan BJ, McMurdie PJ, Rosen MJ, Han AW, Johnson AJA, Holmes SP. 2016. DADA2: high-resolution sample inference from Illumina amplicon data. Nat Methods 13:581–583. doi:10.1038/nmeth.3869 27214047 PMC4927377

[26] Matsuo Y, Komiya S, Yasumizu Y, Yasuoka Y, Mizushima K, Takagi T, Kryukov K, Fukuda A, Morimoto Y, Naito Y, Okada H, Bono H, Nakagawa S, Hirota K. 2021. Full-length 16S rRNA gene amplicon analysis of human gut microbiota using MinION nanopore sequencing confers species-level resolution. BMC Microbiol 21:35. doi:10.1186/s12866-021-02094-5 33499799 PMC7836573

[27] Jimeno R, Brailey PM, Barral P. 2018. Quantitative polymerase chain reaction-based analyses of murine intestinal microbiota after oral antibiotic treatment. J Vis Exp 141. doi:10.3791/58481 30507921

