# Supplementary figures and images for: Identification of beneficial symbiont candidates in commensalism as potential oral gatekeepers
Source: Microbiol Spectr. 2025 Sep 5;13(10):e01588-25. doi: 10.1128/spectrum.01588-25 (PMC12502559; doi:10.1128/spectrum.01588-25)

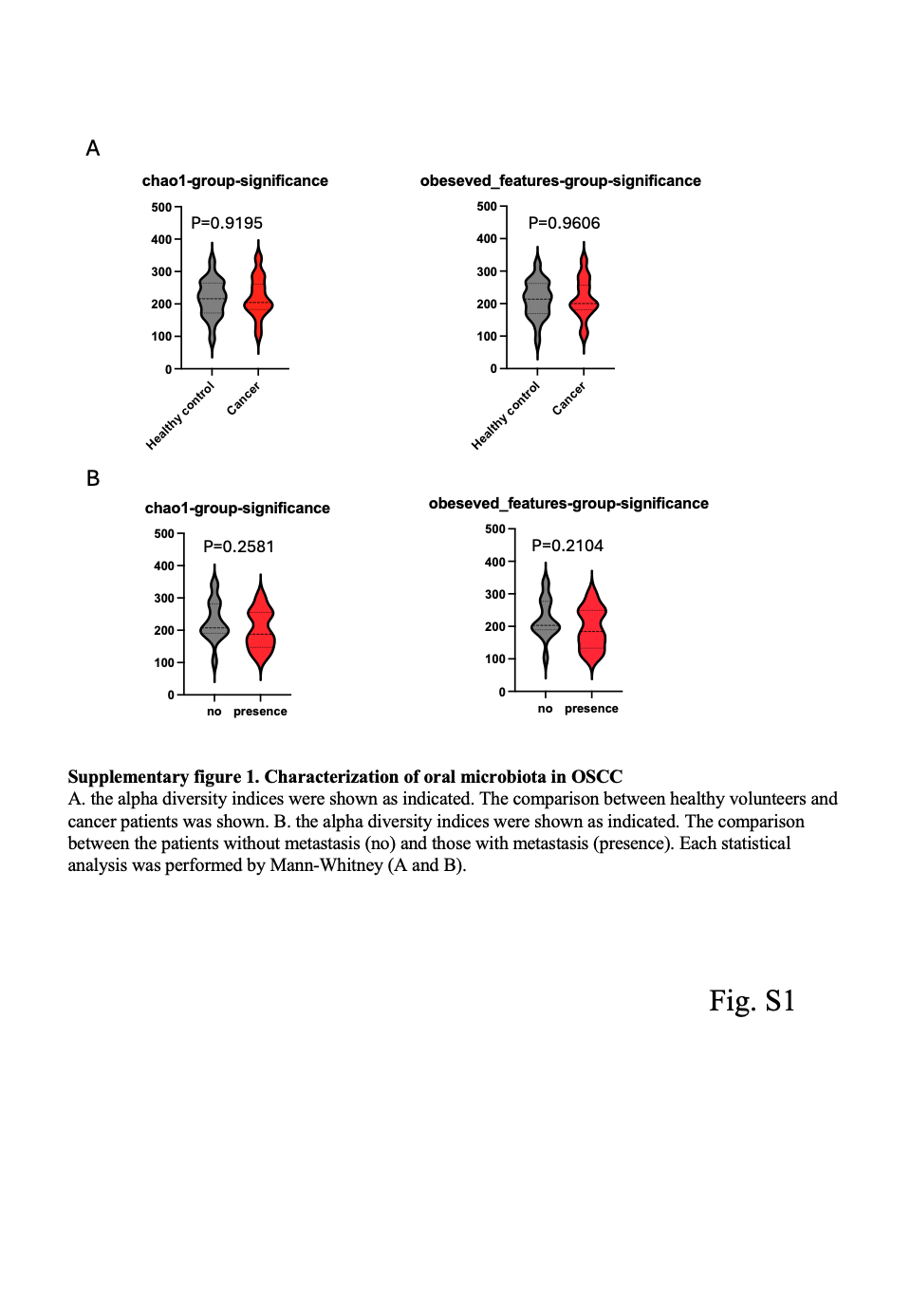

Supplement: Fig. S1 — Characterization of oral microbiota in OSCC. [file spectrum.01588-25-s0001.tiff]

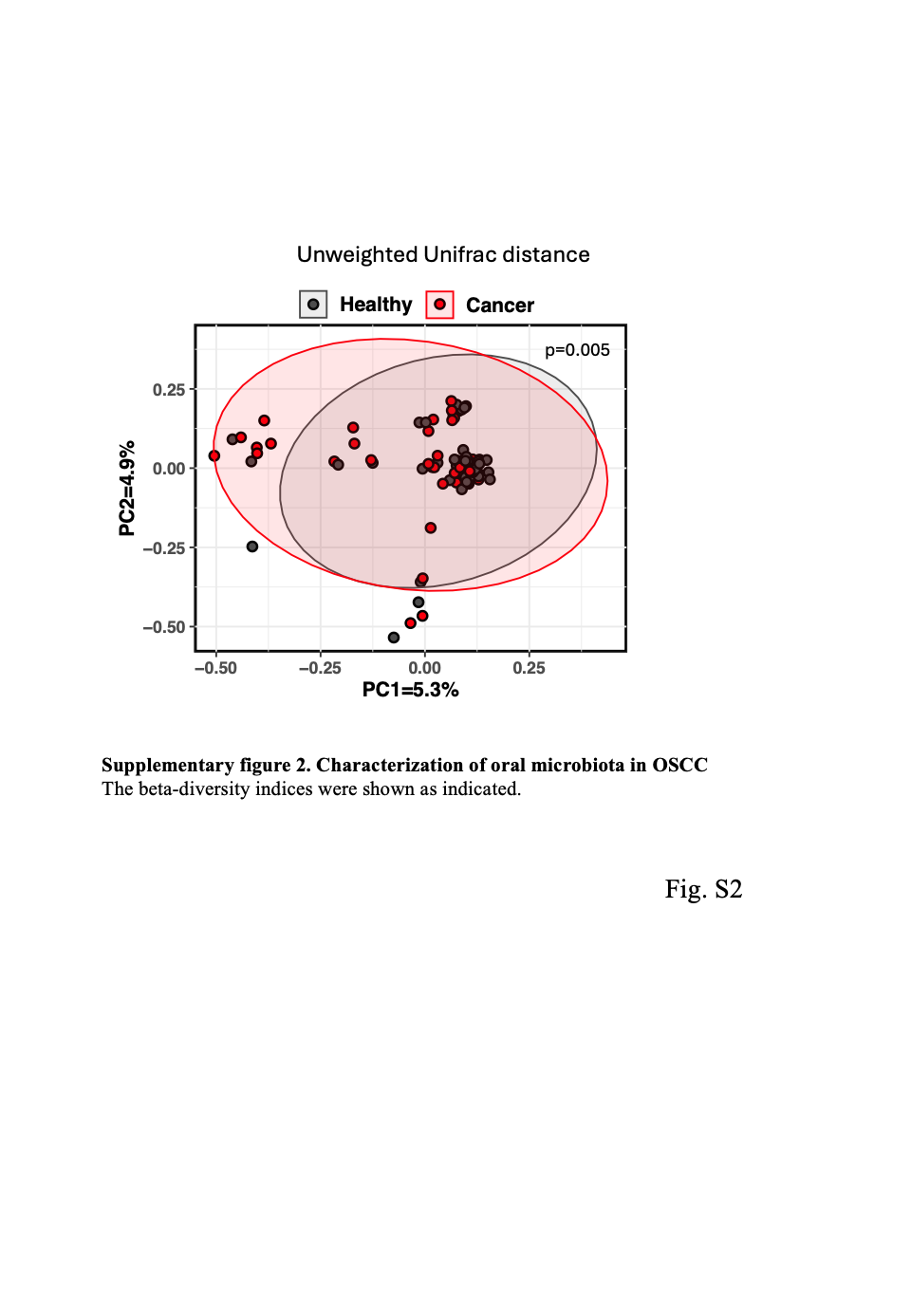

Supplement: Fig. S2 — Characterization of oral microbiota in OSCC. [file spectrum.01588-25-s0002.tiff]
